# Analysis of Learning Influence of Training Data Selected by Distribution Consistency

**DOI:** 10.3390/s21041045

**Published:** 2021-02-04

**Authors:** Myunggwon Hwang, Yuna Jeong, Won-Kyung Sung

**Affiliations:** 1 Intelligent Infrastructure Technology Research Center, Korea Institute of Science and Technology Information (KISTI), Daejeon 34141, Korea; mgh@kisti.re.kr (M.H.); wksung@kisti.re.kr (W.-K.S.); 2 Department of Data & HPC Science, University of Science and Technology (UST), Daejeon 34113, Korea

**Keywords:** learning influence, machine learning, training data similarity, distribution consistency

## Abstract

This study suggests a method to select core data that will be helpful for machine learning. Specifically, we form a two-dimensional distribution based on the similarity of the training data and compose grids with fixed ratios on the distribution. In each grid, we select data based on the distribution consistency (DC) of the target class data and examine how it affects the classifier. We use CIFAR-10 for the experiment and set various grid ratios from 0.5 to 0.005. The influences of these variables were analyzed with the use of different training data sizes selected based on high-DC, low-DC (inverse of high DC), and random (no criteria) selections. As a result, the average point accuracy at 0.95% (±0.65) and the point accuracy at 1.54% (±0.59) improved for the grid configurations of 0.008 and 0.005, respectively. These outcomes justify an improved performance compared with that of the existing approach (data distribution search). In this study, we confirmed that the learning performance improved when the training data were selected for very small grid and high-DC settings.

## 1. Introduction

The performance of artificial intelligence (AI) applications is influenced by machine learning (ML) models and training data. In here, we define that a term “learning influence” means an accuracy of a trained ML model. The development of ML models has received intense attention, and to-this-date, there has been significant progress in their development [1,2,3,4,5,6,7,8,9,10,11,12,13]. To train these models more accurately, high-quality data must be obtained. It is well-known that a larger amount of data has a more positive influence on the learning performance, and an increased amount of time and cost needs to be invested to collect the data [14]. Using data above a certain quantity increases the computational complexity, while the corresponding learning performance improvement is not considerable. As a result, more time, memory, and computational resources are required for learning that affect adversely its cost effectiveness.

A large amount of data and complex models require a considerable amount of time for each epoch that impedes the development of AI. To develop the AI that is executing specific tasks automatically and intelligently, ML experts should be involved for specific jobs, such as data acquisition and preprocessing, model selection, hyper-parameter tuning, optimizer selection, performance matrix selection, and others. These jobs are repeatedly required in the ML process. This is referred to as the human-in-the-loop (HITL) process and the process is repeated until the AI achieves the performance objective [12].

To shorten the HITL process, we propose a method of distribution consistency. This method can be applied to active learning, curriculum learning, and other types of learning that require ML efficiency. This method involves processes that form a two-dimensional distribution of the training data, composes grids on the distribution, and selects a predetermined size of data according to the order of distribution consistency (DC).

Based on a method of data distribution search that was performed in our previous study, we inferred that a data selection process that considers the data density in each grid was effective to some extent. However, this was not applicable to the cases where data less than 40% of the entire data were selected (random selection was better [15].

In the present study, we have developed further the DDS. Accordingly, this study describes the investigation of a method that can have a higher accuracy, contributes to the performance improvement in all selections of different number of data, and shares data selection insights gained from the experiments.

The remainder of this study is organized as follows. Section 2 describes the background research of this study, and Section 3 introduces the DC method with previous work. Its experimental analysis is explained in Section 4. Conclusions are outlined at the end with suggestions for further research in Section 5.

## 2. Background Research

**Figure 1:**
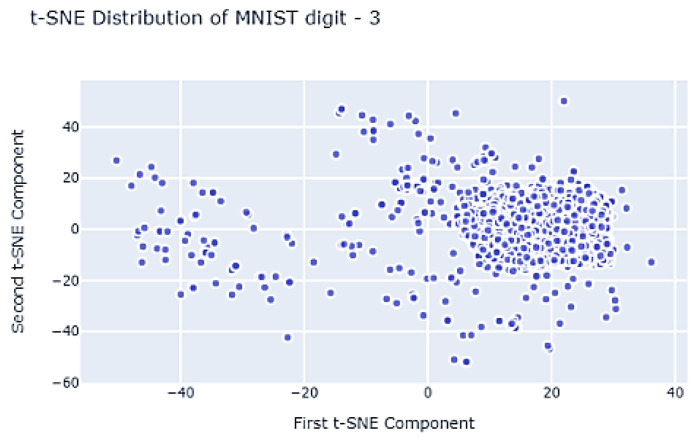
Two-dimensional distribution of MNIST data (digit 3) using t-SNE.

This study was initiated based on the question, “Can we find a method to select training data that contribute more to ML?” To find the answer, we started with the hypothesis that “similar data make a similar contribution to ML.” We found in the previous work that the hypothesis was correct [15]. Figure 1 shows the distribution of the MNIST (*The MNIST DATABASE of handwritten digits: http://yann.lecun.com/exdb/mnist/*) dataset after dimensional reduction using t-distributed stochastic neighbor embedding (t-SNE). Each point corresponds to one image.

As indicated, the data distributed in the neighborhood have similar characteristics. Thus, we could assume that the learning contribution rate of the adjacent data would be similar. In other words, ML would learn diversity from a small number of data in various areas. According to this idea, when data from each class are distributed like in Figure 1, a small percentage of data is selected in the dense area (the actual selected data may be equivalent or higher than the sparse area), and a large percentage of data is selected in the sparse area. From this observation, we proposed a data distribution search (DDS) technique in the work [15] that formed subsets of training data from the data distribution for each class.

To assess the performance of the proposed method, MNIST and CIFAR-10 datasets were used, and the DDS selected subsets of training data with predetermined ratios, i.e., {60%, 50%, 40%, 30%, 20%, 10%, 4%, 3%, 2%}. Finally, we trained classifiers corresponding to the subsets and tested each classifier with a test set (10,000 data).

Based on this test, we could confirm that the subsets selected by DDS yielded superior performance compared with those of random selections. However, the improvement was limited, and worked well only for selections of 60%, 50%, and 40%, in the case of CIFAR-10 (The CIFAR-10 Dataset: https://www.cs.toronto.edu/~kriz/cifar.html).

The key point identified based on these results was that the distribution-based data selection was able to select the core subsets. This study aims to overcome the existing limitation and describe a new method to provide a better selection for core subsets with high contributions to the learning performance.

## 3. Method of Distribution Consistency

This section describes the analysis of the learning influence based on the consistency of the data distribution.

### 3.1. Overview of Research Architecture

**Figure 2:**
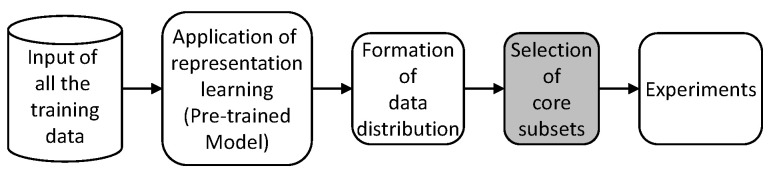
Research architecture.

As shown in Figure 2, the overall architecture of this study is composed of five steps: input of all the training data, application of the representation learning model, formation of data distribution, selection of core subsets, and experiments (classifier that trains and tests).

Additionally the detailed descriptions are as follows.

- Input of all training data: We prepared training data in this step, and used CIFAR-10 which contained real-world images, but its size was very small (32 × 32 pixels). This means that the classification task is not easy with this data [16].
- Application of representation learning model: We applied one of the methods for dimensional reduction such as t-SNE, principal component analysis (PCA), and ISOMAP [11,17,18]. These methods defined core features of simple data like MNIST and reduced their dimensionality well, but this was not the case for complicated data like CIFAR-10 which have high complexity. To overcome this limitation, we employed a pre-trained model that can understand image features. Accordingly, in this study, GoogLeNet (InceptionV3) was used [9,10].
- Formation of data distribution: This step analyzes the high-dimensional data, extracts key features, and generates an *n*-dimensional distribution. In general, this reduction is used to simplify data computation, and facilitate better understanding and visualization. We employed t-SNE and reduced it in two dimensions.
- Selection of core subsets: This is the most important part of this study and is described in detail in Section 3.2.
- Experiments: In this step, we set a convolutional neural network, train the network with each subset of various sizes, and evaluate its classification performance with 10,000 test sets. Furthermore, we compare the performance with previous work and random selection. This is described further in Section 4.

### 3.2. Subset Selection Based on Distribution Consistency

We suggest distribution consistency (DC) as a further developed version of the DDS presented in our previous work [18]. The DDS considered the data distribution of a single (target) class. Specifically, the training data were input to t-SNE to form a two-dimensional distribution. Furthermore, the DDS divided the distribution into a fixed size with grid ratio (*r_g_*), iteratively selected *m* data from dense to sparse grids, and finally formed *n* data. Herein, the set {*r_g_*, *m*, *n*} constituted the hyper-parameter. These steps were repeated within the number of classes, i.e., ten times for MNIST and CIFAR-10.

**Figure 3:**
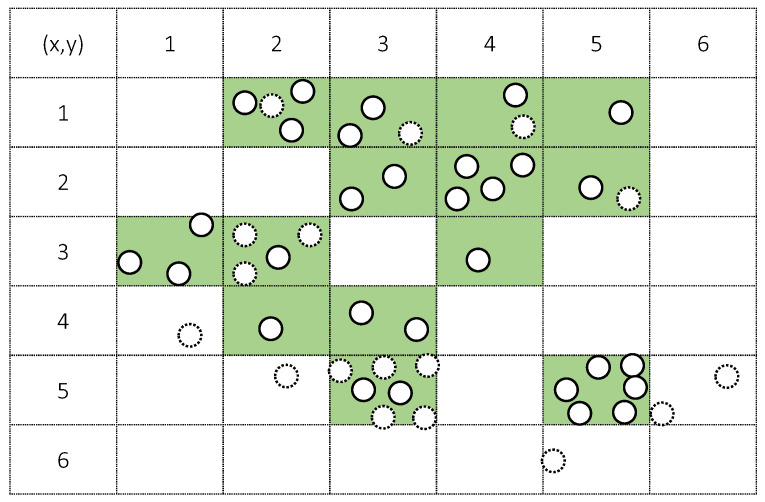
Example of data distribution of a single class and grid formation with 1/6 given for *r_g_* against the total x, y length (solid-line circles: target class, dotted-line circles: other class data).

With the example of Figure 3, the DDS selects the subset from all data on the basis of priority like Table 1 in the case of {1/6, 1, 15} for {*r_g_*, *m*, *n*}.

**Figure 4:**
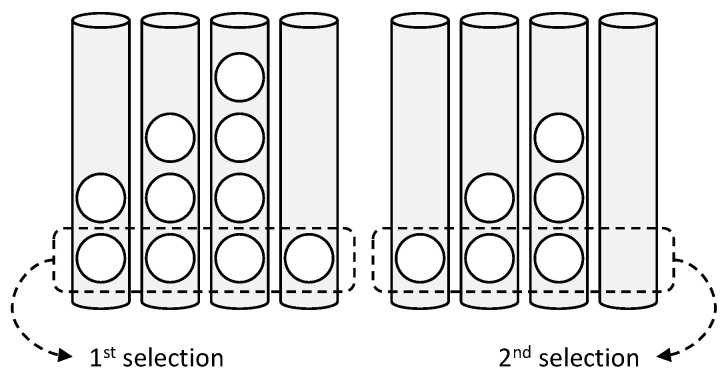
Two-dimensional distribution of MNIST data (digit 3) using t-SNE. Data selection for each step based on the DDS (select m data from each grid sequentially, currently m is 1).

The DDS only considers the distribution of a target class, and thus ignores other class data in each grid. Additionally, *m* data are selected from the high priority to the low priority. In the case in which the priority is the same, one grid is selected randomly. Figure 4 shows this data selection process.

There are 2, 3, 4, and 1 target data in four grids (cylinders in the figure) in the left of Figure 4. Because *m* is equal to one, one datum is selected from all grids in the first search and the fourth grid becomes empty. Thus, in the second search, one datum is selected from the three grids. This process is run continually until *n* data are selected in total.

On the basis of the DDS method, we considered the DC of data for further development. In other words, it is a method used to assign weights to the grids by measuring the consistency ratio of the target class data. This is measured by (1).

(1) $$weight_{consistency}=\frac{n_{target}}{n_{total}}$$

where, the division of *n_target_* by *n_total_* represents the ratio of the target data contained in the grid. The purpose of this study was to select the ones with high DC (*weight_consistency_*) to investigate the learning influence. However, we also examined the cases that selected the subset according to a descending order, called low DC (= 1 − *weight_consistency_*). In Table 2, the overall process of data selection is shown using the distribution represented in Figure 3.

**Figure 5:**
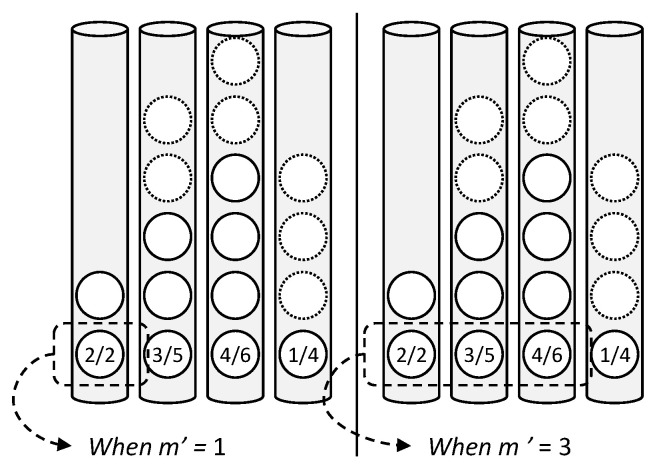
Data selection strategy based on distribution consistency (DC).

Table 2 shows the conceptual examples of data selection based on the high DC and low DC simultaneously. The strategy in which data are selected is different from the DDS. This is summarized in Figure 5.

Data selection started with the input of a set combination {*n*, *r_g_*, *m’*}, namely, with the number of subset data, the grid ratio, and the number of grids selected in a single calculation, respectively. Herein, *m’* is different from *m* (of DDS), which denotes the number of data selected from each grid. As shown in Figure 5, DC selects one grid (*m’* = 1) or three grids (*m*’ = 3) according to the DC weight iteratively until *n* number of data are filled. The data in each grid is selected randomly, and is the same as the DDS.

Using the DC weight proposed in this study, we selected a wide range of subsets, and experimented with the data regarding their learning performance.

## 4. Experimental Analysis

### 4.1. Experimental Environmental

The experiment was conducted as follows.

- Data: this method can be applied to every type of image. However, in this case we focused on CIFAR-10 only that contained real-world images with very small sizes and on difficult mage classification tasks [19].
- Training set configuration: CIFAR-10 is composed of ten classes, and each class consists of 5000 and 1000 images for training and testing, respectively. Herein, 3000, 2500, 2000, 1500, 1000, 500, 250, 200, 150, and 100 data from the original set were selected to form the training subsets, and the entire test set was used for all classification tasks. The selection of data was based on the three criteria: random selection, the high-DC weight, and the low-DC weight.
- Classification model configuration: The CIFAR-10 dataset is considerably different from that obtained from MNIST and requires complex convolutional neural network architecture. Therefore, in this study, a convolution model with 14 hidden layers was configured, as shown in Table 3. By using this model, a test accuracy of approximately 0.7846 (average value of results of five iterations—the same operation was applied to all subsequent experiments) was achieved when the classification model was trained with the entire training data.

We compared the test accuracies of the trained model with the subsets selected by three different ways.

### 4.2. Test Accuracy of Random Selection

**Figure 6:**
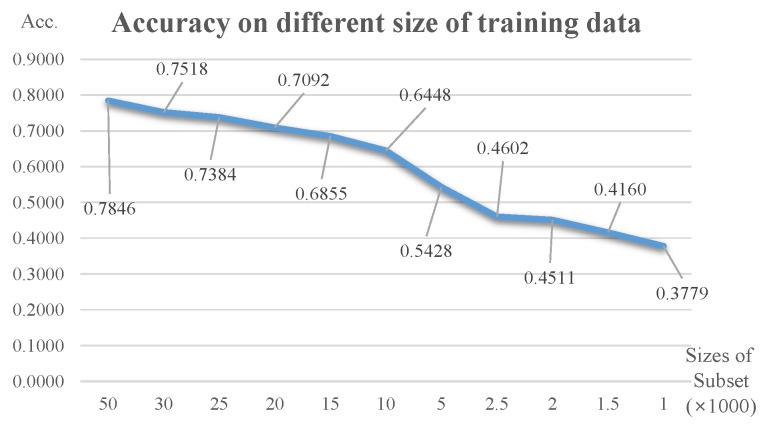
Learning performance of randomly selected data.

First, we measured the learning performance of the subsets selected randomly. As mentioned in Section 4.1, we prepared five sets of each subset by random selection from the entire CIFAR-10 dataset and the subsets consisted of 30,000, 25,000, 20,000, 15,000, 10,000, 5000, 2500, 2000, 1500, and 1000 data respectively. We trained the model of Table 3 with the prepared data, and tested each model with 10,000 test set. We calculated their accuracies (correct answer rates) on average and Figure 6 summarizes the result.

An accuracy of 0.7846 was obtained with the entire dataset (50,000 in total), and its value decreases to 0.7518, 0.7092, and to smaller values with fewer training subsets.

It is natural that learning performance improves as the number of data increases, but the degree of improvement is not directly proportional to the number of data. That is, randomly increasing the number of data or creating a model by using the entire dataset from the beginning may be costly in terms of computation time, while its performance improvement is limited. This is one of the major reasons for which we developed a data selection method that can positively influence the learning performance. There is a Selection via Proxy (SVP) method which is one of the latest research work for core data selection [19]. The SVP compared its performance with random selection. From this aspect, we also aimed to derive superior performance compared with the case of randomly selected data.

### 4.3. Learning Performance according to Distribution Consistency

To investigate the effect of DC proposed in Section 3.2, various combinations of {*r_g_*, *mʹ*, *n*} were formed as follows:

- *r_g_*: {0.5, 0.25, 0.2, 0.125, 0.1, 0.05, 0.04, 0.025, 0.02, 0.01, 0.008, 0.005}. These are decimal numbers that can be divided by unity without leaving a remainder. Grids with sizes of 2 × 2 are formed in the case of 0.5 and of 200 × 200 in the case of 0.005.
- *mʹ*: {1} means that *m’* is equal to one. Up to this now, we only considered how effective the distribution consistency and the grid ratio are.
- *n*: {30,000, 25,000, 20,000, 15,000, 10,000, 5000, 2500, 2000, 1500, 1000}. This sequence is the same as the random selection described in Section 4.2.

**Figure 7:**
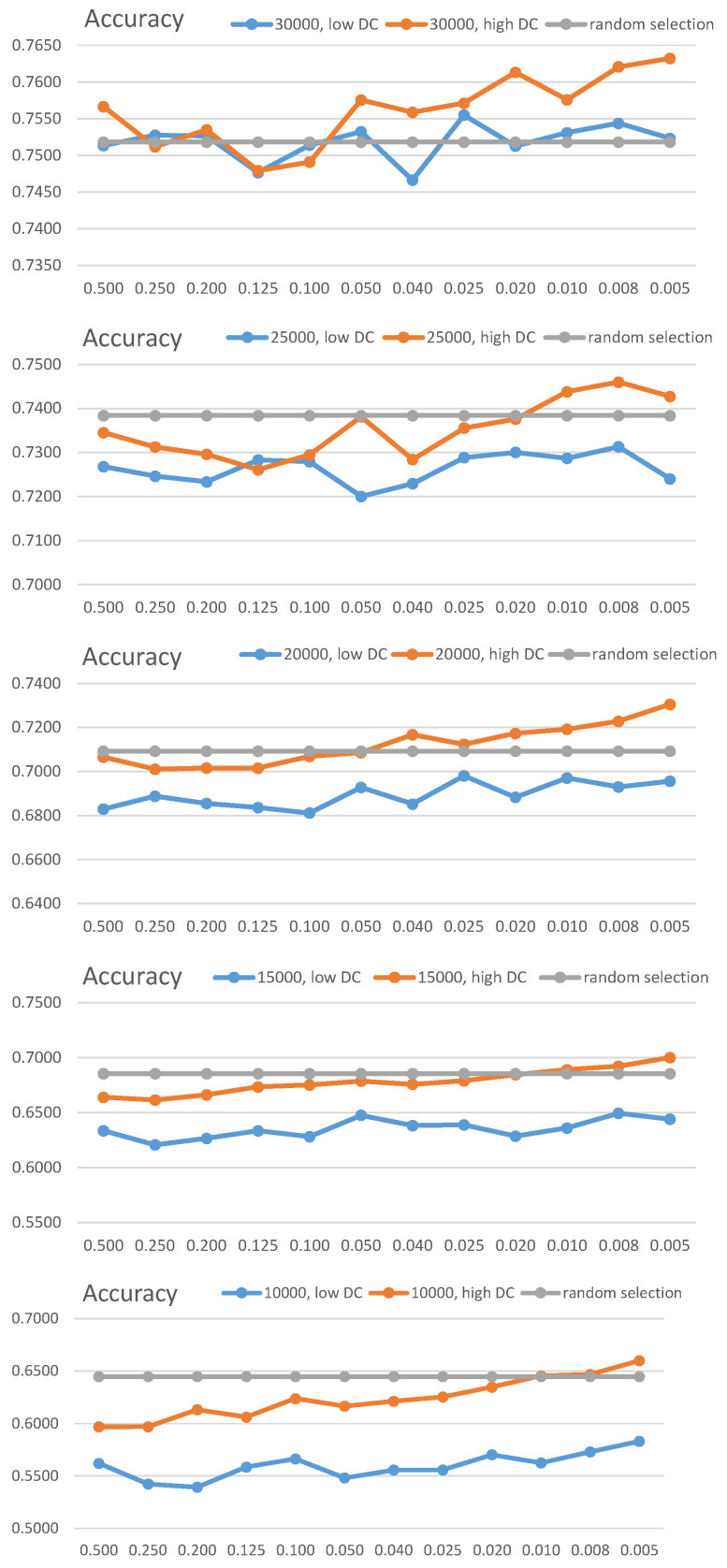

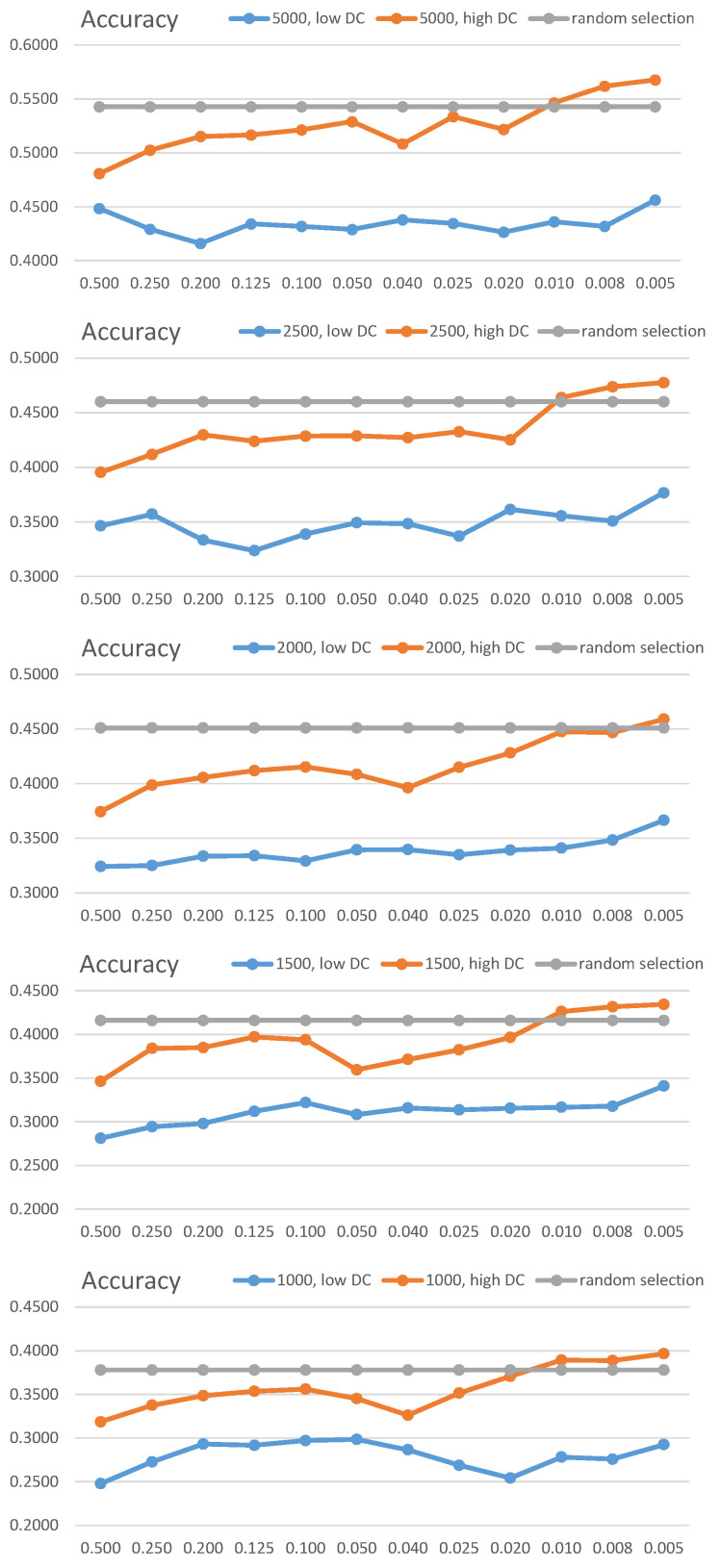
Classification accuracy on distribution consistency and data size (the x-axis represents the value of *r_g_*).

In these configurations, we trained the classification model with the data obtained from each combination, and applied the model to the test set. This process was iterated five times to calculate the average accuracy. A summary of the results is shown in Figure 7.

In Figure 7, the results of training and tests are shown using datasets of size *n* from the top to the bottom. The orange line indicates the accuracies of high DC, the blue line is the low DC, and the gray line means the random selection. The overall test results, based on these combinations, indicate that the data selected based on the high DC positively influence the increases in the test accuracy. In addition, it can be observed that the detailed subdivision of the grids increases the performance. In the case of CIFAR-10, the complexity of the data is high, and it is thus difficult to show outcomes superior to the learning performance of randomly selected data. However, we could confirm that superior accuracy was achieved when the grid ratios of 0.008 and 0.005 were used, compared with the case of random selection.

**Figure 8:**
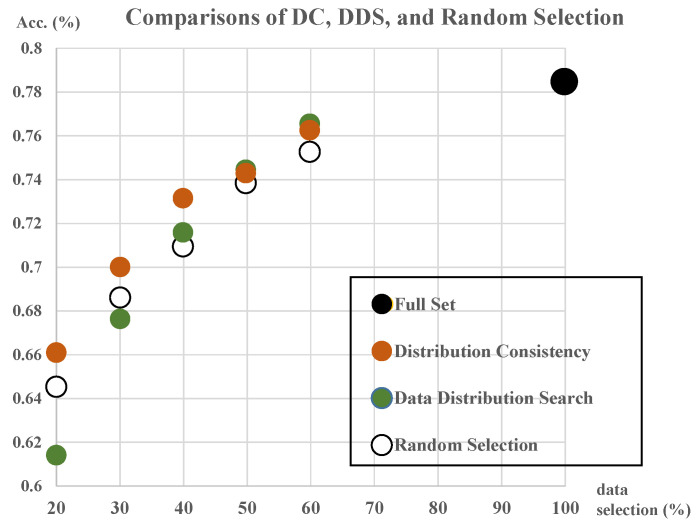
Accuracies of data subsets selected based on high DC (*r_g_* = 0.005), DDS (the best case), and random selection.

We compared the accuracies with the random selection and the DDS. Figure 8 shows the comparison outcomes. The DDS shows the best accuracy for a 60% selection but it is not the case in others, even when it achieved the worst performance (selection of data less than 40%). Conversely, the DC achieved stable accuracies within the entire selection range.

In summary, the implication of this study is associated with the fact that we can enhance the learning performance when we select the data based on the detailed grid ratio and the priority depending on the high DC values. We could confirm this with Figure 7 that consistently shows the better accuracies in the combination of the detailed grids (specifically, 0.008 and 0.005) and high DC. Moreover, the high DC attained more stable data selection, as shown in Figure 8, than that of DDS. DDS showed the best accuracies in two cases (60% and 50% data selection) but decreased drastically in lesser data selection. On the other hand, the high DC method has the stable and the best performance in general.

To identify the reason for this implication, we investigated the distribution of selected data based on high and low DCs. Table 4 and Table 5 show the data distributions for one class. Each distribution contains all the data (5000 orange and blue points), wherein orange points denote the selected data and blue points do not. Herein, from the difference in the DC, it can be observed that the cases associated with high DC select data from a dense area, and the cases associated with low DC select data from a sparse area. Considering that high DC exhibits superior performance, this finding is the opposite of the premise of prior research [16] in which the data distributed in similar areas were found to have similar contributions to the learning performance. In addition, we can check that as the grid ratio decreases and becomes more refined, the area in which data are selected is expanded. These results empirically demonstrate that data selection from a wide area with a high DC can positively contribute to the learning performance.

### 4.4. Time-Efficiency Analsysis

**Figure 9:**
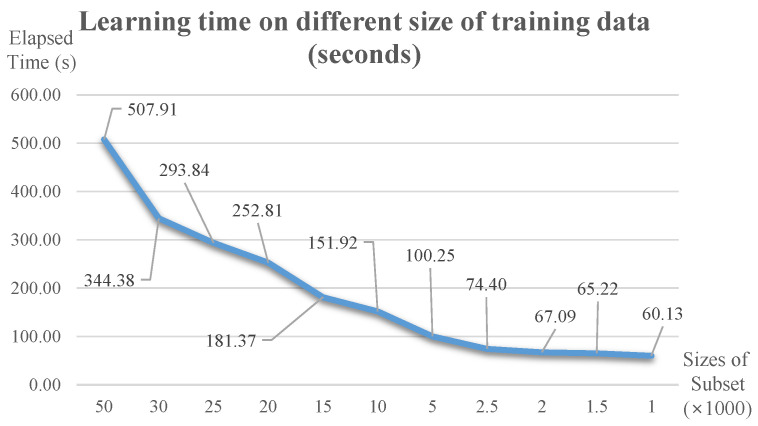
Learning time according to training data size (Unit: s, using NVIDIA GeForce RTX 2080Ti).

Because a routine for selecting core subsets for ML is added prior to the classifier, additional time is required for computation. First, we measured the elapsed time for learning with different numbers of data, and Figure 9 shows the result. Evidently, more time is needed when more data are available, but the performance does not improve considerably (refer to Figure 8). This means that the time for HITL can be shortened by starting with a small amount of data to create an optimal ML model. Furthermore, the method can be utilized for various applications such as data selection for active learning, curriculum learning, and others. These points indicate why it is important to form core subsets for efficient training.

The time for data selection is added to the aforementioned classifier, and the DC method is expected to expend approximately 13.28 s, as shown in Table 6. The selection process is performed just once before entering the stage of ML. This means that it is independent of the HITL process which requires the maximum amount of time and cost to create AI. In addition, it can be regarded that the added time is not large compared with the time taken for model training, especially in view of the fact that the method demonstrates better performance compared with random selection.

## 5. Conclusions

We suggested a method that selected core subsets to contribute positively to the learning performance. For this purpose, we investigated the effects on test accuracy according to the DC of training data. Specifically, we employed the InceptionV3 model to interpret complex images, and the t-SNE method to reduce to low *n*-dimensions, especially to two-dimensional planes for easy understanding. Furthermore, we divided the plane into grids at fixed ratios, and calculated weights with the use of high DC and low DC of each grid. As a result, we confirmed that the learning performance could be better when training data were selected in conditions in which the grid ratio was less than 0.008, and the distribution consistency was high. In addition, this selection achieved the best performance that was more stable than those of DDS and random selection.

ML is an AI development tool and it accompanies an HITL process in which humans are repeatedly involved until they reach the desired performance in the development of automatic and intelligent software. It is almost impossible to reach the targeted performance in a few trials. Instead, additional data collection, data preprocessing, model selection, and hyper-parameter tuning are needed, but these require significant amounts of time and cost. The method proposed in this study is related to the data collection in the HITL and can contribute to shortening the HITL time to derive the optimal model with a smaller amount of data. Moreover, the results of this study present the criteria for selecting training data that improve the learning performance, rather than using indiscreetly large amounts of data when additional data are needed, including active learning. In addition, the findings of this study can be utilized in the development of a strategy for the selection of training data in curriculum learning to quickly reach the global minimum point of the loss function when training is performed with the entire set of acquired data.

Based on this viewpoint, numerous additional studies ought to be conducted. We will investigate the performance change by setting the diversity of *m’* values by changing various representation learning models and dimensional reduction methods, and by applying newly improved selection methods. Furthermore, we will use various benchmarking datasets. Overall, we will apply the selection methods for active and curriculum learning.

## Figures and Tables

**Table 1:** Data distribution search (DDS)-based selection process.

No.	Grid Position (*x*, *y*)	Number of Target Data	Priority
1	2, 1	3	3
2	3, 1	2	4
3	4, 1	1	5
4	5, 1	1	5
5	3, 2	2	4
6	4, 2	4	2
7	5, 2	1	5
8	1, 3	3	3
9	2, 3	1	5
10	4, 3	1	5
11	2, 4	1	5
12	3, 4	2	4
13	3, 5	2	4
14	5, 5	5	1

Order of selection: First search {14, 6, 1, 8, 2, 5, 12, 13, 3, 4, 7, 9, 10, 11}, second search {14}.

**Table 2:** Data selection process based on high-distribution consistency (DC) and low DC.

No.	*x*, *y*	Data Number		Weights		
		Target	Others	1st	2nd	3rd
1	2, 1	3	1	3/4	2/3	1/2
2	3, 1	2	1	2/3	1/2	-
3	4, 1	1	1	1/2	-	-
4	5, 1	1	0	1/1	-	-
5	3, 2	2	0	2/2	1/1	-
6	4, 2	4	0	4/4	3/3	…
7	5, 2	1	1	1/2	-	-
8	1, 3	3	0	3/3	2/2	1/1
9	2, 3	1	3	1/4	-	-
10	4, 3	1	0	1/1	-	-
11	2, 4	1	0	1/1	-	-
12	3, 4	2	0	2/2	1/1	-
13	3, 5	2	5	2/7	1/6	-
14	5, 5	5	0	5/5	4/4	...

High-DC-based selection: 4 → 5 → 6 → 8 → 10 → 11 → 12 → 14 → 5 → 6 → 8 → 12 → 14 → 6 → 8. Low-DC-based selection: 13 → 13 → 9 → 3 → 7 → 2 → 2 → 1 → 1 → 1 → 4 → 5 → 6 → 8 → 10.

**Table 3:** Classification model setting.

Layer	Setting
Input	Conv2D (32,(3,3)), ReLU), he_uniform
Hidden-1	Conv2D (32,(3,3)), ReLU, he_uniform
Hidden-2	MaxPooling2D(2,2)
Hidden-3	Dropout (0.2)
Hidden-4	Conv2D (64,(3,3)), ReLU, he_uniform
Hidden-5	Conv2D (64,(3,3)), ReLU, he_uniform
Hidden-6	MaxPooling2D (2,2)
Hidden-7	Dropout (0.2)
Hidden-8	Conv2D (128,(3,3)), ReLU, he_uniform
Hidden-9	Conv2D (128,(3,3)), ReLU, he_uniform
Hidden-10	MaxPooling2D (2,2)
Hidden-11	Dropout (0.2)
Hidden-12	Flatten ()
Hidden-13	128 Dense Layer, ReLU, he_uniform
Hidden-14	Dropout (0.2)
Output	10 Dense Layer, Softmax

Batch size = 64, epochs = 50.

**Table 4:** Data distribution selected by high-DC (3000 Data of Class Number 0).

r_g_	Data Distribution
0.125	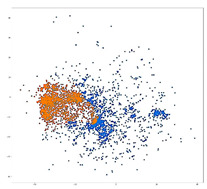
0.100	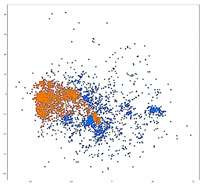
0.020	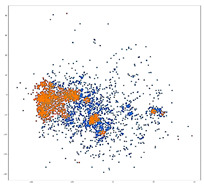
0.008	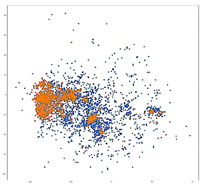
0.005	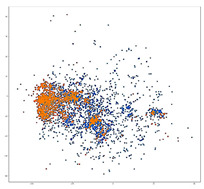

**Table 5:** Data distribution selected by low-DC (3000 data of class number 0).

r_g_	Data Distribution
0.125	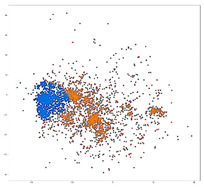
0.100	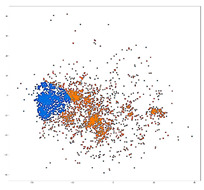
0.020	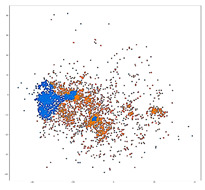
0.008	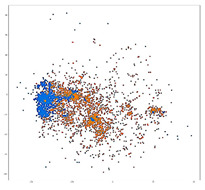
0.005	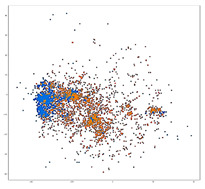

**Table 6:** Time required for data selection (Grid Ratio: 0.005).

Number of Data	Elapsed Time for Selection (s)
30,000	12.550
25,000	13.600
20,000	13.697
15,000	13.465
10,000	13.409
5000	13.334
2500	13.266
2000	13.207
1500	13.200
1000	13.112

Computer: Windows 10, 64 GB memory, Intel Core processor i9-9980XE 3.00 GHz.

## Data Availability

Data sharing not applicable.
